# Microscopic identification and in vitro activity of *Agastache rugosa* (Fisch. et Mey) from Xinjiang, China

**DOI:** 10.1186/s12906-017-1605-7

**Published:** 2017-02-07

**Authors:** Haiyan Gong, Shaoyu Li, Lijuan He, Rena Kasimu

**Affiliations:** 1 Postdoctoral research station of college of pharmacy, Xinjiang Medical University, Xinshi District, No. 393 Xinyi Road, Urumqi, Xinjiang People’s Republic of China; 2 The Fifth Affiliated Hospital of Xinjiang Medical University, Xinshi District, No. 118 Henan West Road, Urumqi, Xinjiang People’s Republic of China; 3 The Third Affiliated Hospital of Xinjiang Medical University, Xinshi District, No. 789 Suzhou East Road, Urumqi, Xinjiang People’s Republic of China; 4 Xinjiang Medical University, Xinshi District No. 393 Xinyi Road, Urumqi, Xinjiang People’s Republic of China

**Keywords:** Microscopic identification, *Agastache rugosa*, Antibacterial effect, Cytotoxicity

## Abstract

**Background:**

*Agastache rugosa* is well-known as a common traditional Chinese medicine, which have relieving summer-heat, analgesic and antipyretic effects, have long been used as folkloristic remedy in the treatment of several infectious diseases, anti-inflammatory, and for its antibacterial properties. Considering the lack of available data on the morphology, anatomy and in vitro activity of *A. rugosa,* the goal of the present study was to carry out the microscopic identification of its aerial parts and in vitro activity research as a contribution to the quality control and reasonable utilization involving *A. rugosa.*

**Methods:**

The present study was (a) to describe the microscopic identification with usual light and scanning electron microtechniques of *A. rugosa*, collected from Xinjiang Region; (b) based on previous research on the essential oil constituents among different parts of *A. rugosa* from Xinjiang by GC-MS method, to evaluate its antibacterial effect and cell viabilitity assay.

**Results:**

The microscopic identification of botanical material showed some typical structure. The essential oils from the dried flower (EOF) and leaves (EOL) of *A. rugosa* were 0.29% and 0.57% (w/w), respectively. The in vitro antibacterial activities showed strong inhibition against *S.aureus, E. coli* of EOF; strong inhibition against *E. coli* of EOL. Based GC-MS analysis, the MTT assay showed a dose and time-dependent increase in damage for gastric cancer cell line SGC-7901.

**Conclusions:**

The results of this work, based on an extensive analytical characterization of the EOF and EOL chemical composition, compared with other origins, showed *A. rugosa* possessed antibacterial and cytotoxicity properties activities, which need much additional work to open up new biomedical application of these components.

## Background

**Fig. 1:**
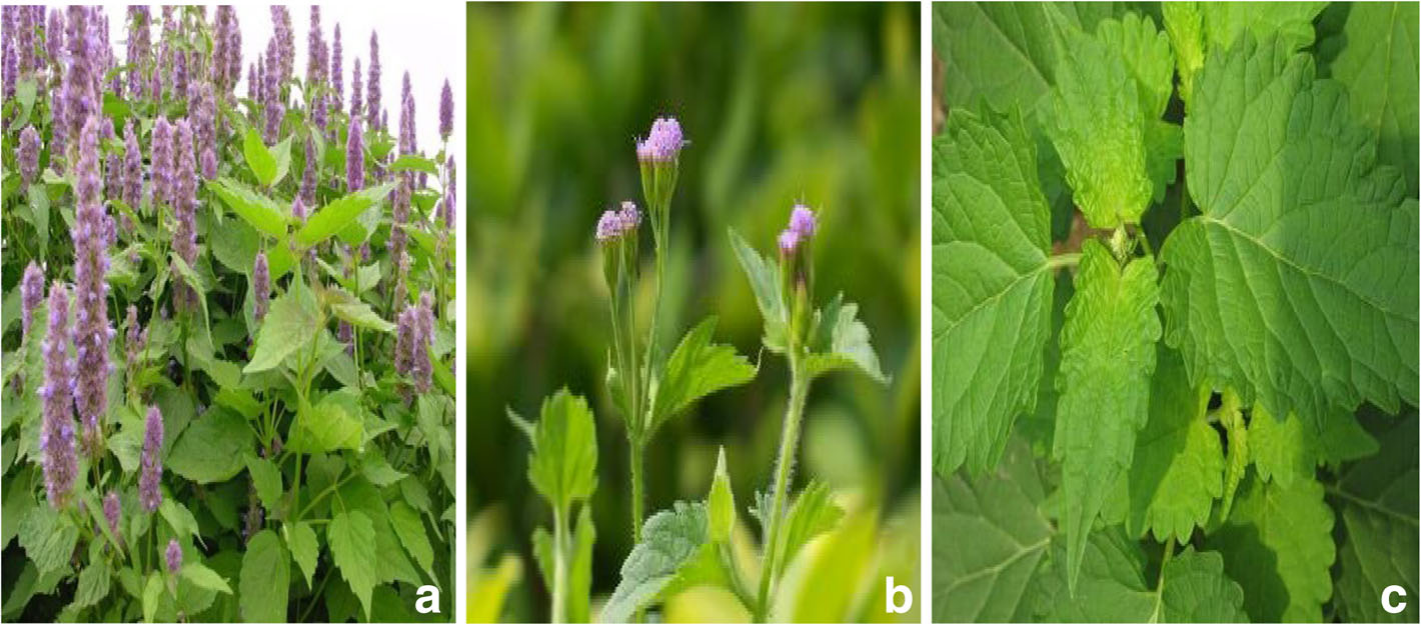
The photo of Agastache rugosa from Xinjiang, China

*Agastache rugosa* (Fisch. et Mey) commonly known as Korean mint, purple giant hyssop, Indiana mint, and the wrinkled giant hyssop [1], which belongs to Lamiaceae family and widely grows in various regions of Xinjiang (Fig. 1a). As an edible plant, it is used as a herbal medicine for anxiety, nausea, bacterial infections, or gas in folk medicine. *A. rugosa* is one of the 50 fundamental herbs, which is known as huò xiāng, has antifungal, antibacterial, carminative, and antipyretic properties [2]. As a fungicide, this plant is used to prevent fungus from growing on potato crops [3]. Some studies reported that *A. rugosa* have antioxidant activity [4] and anti-HIV integrase action [5]. *A. rugosa* constitutes one of the most important renewable sources of natural remedy for the populations of the Xinjiang region. In China it is a representative in Huoxiang Zhengqi Liquid, which is family essential medicine. Notwithstanding various terpene compounds were isolated from *A. rugosa* and pharmacological studies have been done, few studies focus on *A. rugosa* derived from Xinjiang region. An accurate identification of the investigated species is remarkable required for pharmacognostic purposes. So morpho-anatomical studies are essential tools to provide low cost and reliable data, which can be used as the first parameters for medicinal plants quality control. Considering the lack of available data on the morphology, anatomy and in vitro activity of *A. rugosa,* the goal of the present study was to carry out the microscopic identification of its aerial parts and in vitro activity research as a contribution to the quality control and reasonable utilization involving *A. rugosa.*

## Methods

### Plant material

The aerial part of *A. rugosa* were cultivated on Liyu mountain in Urumqi of Xinjiang, China by our research group, and collected in September 2010, Authentication of the species was carried out by the examination of aerial parts, including its flower, stem and leaves by Traditional Chinese Medicine Ethnical Herbs Specimen Museum, Yonghe Li. A voucher specimen (No. TCMEHSM 2010-352) was deposited in the herbarium of Xinjiang Medical University.

The plant material was fixed in FAA 70 [6] and kept in 70% ethanol solution (v/v) [7]. This material was sectioned by hand to obtain semipermanent and permanent slides for microscopic studies. Photos were taken using light microscope with different magnifications.

### Essential oil and extracts isolation

The flower and leaves of *A. rugosa* were separated and dried in shadow at room temperature, submitted (100 g of each) to hydrodistillation with 1 L of distilled water in a Clevenger-type apparatus for 6 h. Then collected the oils and dried with anhydrous sodium sulfate prior to analyze, measured, transferred to glass flasks and stored at 4°C.

The aqueous extracts and alcohol extracts of different parts (flower, leave) were obtained with reflux method and ultrasonic extraction, respectively. Than obtained the crude dried extracts by using a rotary evaporator and freeze dryer. The dried extracts were stored at -20°C until used.

### Biological activity: in vitro studies

#### Test organisms

Organisms contain *Staphylococcus aureus* (ATCC 25923)*, Escherichia coli* (ATCC 25922), which were maintained by serial sub-culturing every day on nutrient agar slants and incubating at 37°C for 18-24 h. The cultures were stored under refrigerated condition. The antifungal activity of the plant was tested against *Candida albicans* (ATCC 10231).

Positive control: Penicillin (Zhongnuo Pharmaceutical Institute Company, H13021634) was served as positive control to determine the sensitivity of *S. aureus* tested. Gentamycin (Zhenzhou Linrui Pharmaceutical Co. Ltd, H41020318) was served as positive control to determine the sensitivity of *E. coli* tested. Fluconazole (Tianjin Pharmaceutical Group Xinzheng Co. Ltd, 100108) was served as positive control to determine the sensitivity of *C. albicans* tested.

#### Antibacterial and antifungal activity

The inhibitory potential of EOF, EOL, aqueous extracts and alcohol extracts of different parts (flower, leaves) against growth of three different bacterial was assessed of minimum inhibitory concentration (MIC) and minimum bactericidal concentration (MBC) using broth microdilution techniques following Clinical and Laboratory Standard Institute methods [8]. The essential oils, aqueous extracts and alcohol extracts were added aseptically to sterile melted Mueller Hinton Broth medium (Sabouraud’s Borth medium for *B. albicans*) to produce the concentration range of 5.25-336 μg/ml for EOF, range of 4.72-302 μg/ml for EOL, range of 3.125-2000 mg/ml for aqueous extracts, range of 1.563-100 mg/ml for alcohol extracts. For the determination of MIC, MBC. Standard reference antibiotics (penicillin, gentamycin, fluconazole) were used as positive control. The MIC was defined as the lowest concentration that completely inhibited growth of the organism, as detected by the unaided eye after incubation for 24 h. The MBC was defined as the lowest concentration at which no microorganism growth was detected on the agar plate. *S. aureus* and *E. coli* were incubated at 37°C, and *C. albicans* was incubated at 25°C.

### Cell viability assay (MTT)

**Fig. 2:**
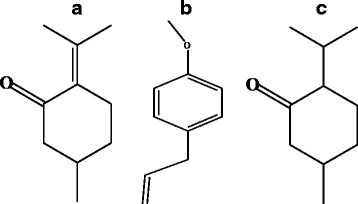
Chemical formula of Pulegone (A), Estragone (B), P-menthan-3-one (C)

The cytotoxicity was measured by the MTT assay using gastric cancer cell line SGC-7901 cells damage induced by essential oils from *A. Rugosa,* which used 5-fluorouracil (5-F) as the positive control. The effect of the plant extracts on the proliferation of the SGC-7901 cells were determined in 96-well tissue culture plates. A MTT colorimetric method based on the reduction of a tetrazolium salt was used according to Kairo et al. [9] with modification of Walencka et al. [10], as followed: after incubation SGC-7901 cells suspensions were added in well per 100 ul (3.44 × 10^7^/well). Based on previous research on the essential oil constituents among different parts of *A. rugosa* from Xinjiang by GC-MS method, the EOF showed pulegone (34.1%), estragole (29.5%), p-Menthan-3-one (19.2%), the EOL showed p-Menthan-3-one (48.8%), estragole (20.8%), as the main components [11]. According to the assay of chemical composition, apply the pulegone (Fig. 2a, Chemical formula, CAS: 89-82-7) and estragole (Fig.2b, Chemical formula, CAS: 140-67-0) as standard substances which were the main active compounds in the EOF and EOL of *A. rugosa.*

Then added different samples: EOF (S1), EOL (S2), estrogole (S3), pulegone (S4) and 5-F in 96-well plates per 10 ul, respectively. Incubating at 37°C, 5% CO_2_ for 24 h, 48 h, 72 h, then add 10 μl of 5 mg/ml MTT (Sigma-Aldrich, Switzerland) in Hank's Balanced Salt Solution (HBSS, Gibco, Life Technologies, Switzerland). After incubate 4 h, the medium was replaced with 200 μl of DMSO (dimethyl sulfoxide) to stop the reaction and lyse the cells. Uninfected SGC-7901 cells served as 0%-damage control and wells containing medium alone were used for background correction. Absorbance of 200 μl of the solution was measured at 560 nm. Damage was calculated using the following formula: 1− (A_560_ of test well/A_560_ of 0%-damage control well).

1 control and 4 experimental groups were run in triplicate for cellular viability analysis by the MTT assay.

### Statistical analysis

All experiments were performed in quadruplicates. A comparative analysis of means was performed using the analysis of variance (ANOVA) and Tukey’s multiple comparison test (*P < 0.05*). Statistical analyses were performed using the SPSS ver. 18.0 software (SPSS Inc., USA).

## Results and discussions

### Microscopic identification

Morphological analysis of *A. rugosa* (Fig. 1a, 1b) reveals stem range from 0.5 to 1.3 m, four prism with 0.6-0.8 mm in diameter, upper being ultrashort floss without for lower. Leave opposite and varying from heart-shaped oval to oblonglanceolate in form and measuring 3.5-11 cm in length and 1.5-6 cm in width, rounded or heart-shaped base, saw-toothed margin, back with puberulent and glandular punctuate. With 1-3.5 cm in length of petiole (Fig. 1c).

**Fig. 3:**
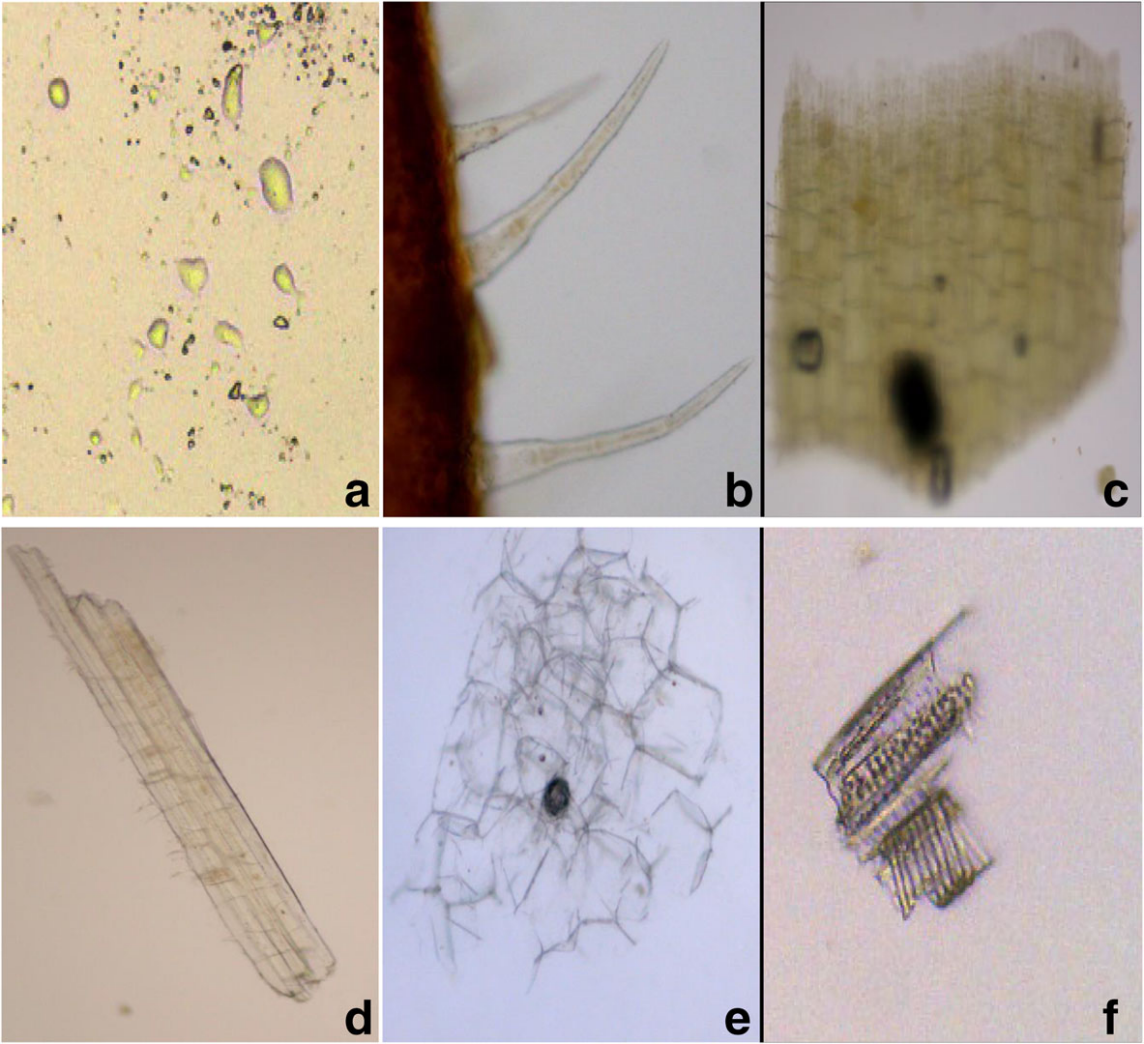
The microscopic identification of *Agastache rugosa* **a**. oil cells; **b**. non-glandular trichomes; **c**. corolla epidermal cells; **d**. wood fiber; **e**. phloem parenchyma cells; **f**. catheter

The microscopic identification of the plant revealed the presence of oil cells, non-glandular trichomes, corolla epidermal cells, wood fiber, phloem parenchyma cells, catheter in flower and leaves powder (Fig. 3).

The results relative to the organoleptic characteristics and to the physical-chemical descriptors of the EOF and EOL are reported in Table 1. The yields of essential oils from flower, stem, leaves were 0.29%, 0.02%, 0.57%, respectively. The data showed the essential oil yields from stem was low, so made the in vitro experiment impossible.

**Table 1:** Physical descriptors of the EOF and EOL of *A. rugosa*

Descriptors	Experimental evaluation	
	Flower	Leave
Color	presented light yellow	brilliant yellow
Odor	powerful spicy odor	stronger aromatic odor
Yield (w/w, %)	0.29	0.57
Specific gravity (g/mL)	0.840 ± 0.001	0.755-0.760
Refractive index (t = 25 °C)	1.1231 ± 0.003	1.1322-1.1400

### Antimicrobial activity

The antibacterial activity results of EOF, EOL, aqueous extracts and alcohol extracts of *A. rugosa* were summarized in Tables 2−4. With the broth dilution method, the MIC values of EOL and EOF were in the range of 9.4-37.8 μg/ml, 21-31.5 μg/ml, respectively. The MBC values of EOL and EOF were in the range of 18.9-75.5 μg/ml, 42-63 μg/ml, respectively. Tables 3−4 showed the aqueous extracts and alcohol extracts from flower and leaves of *A. rugosa* have a certain antibacterial activity.

**Table 2:** Antimicrobial activity of EOL and EOF of *A. rugosa*

Organisms	EOL		EOF		Penicillin		Gentamycin		Fluconazole	
	MIC^a^	MBC^a^	MIC^a^	MBC^a^	MIC^a^	MBC^a^	MIC^a^	MBC^a^	MIC^a^	MBC^a^
*Staphylococcus aureus*	37.8	75.5	21.0	42.0	30	60				
*Escherichia coli*	9.4	18.9	21.0	42.0			100	200		
*Candida albicans*	28.0	57.0	31.5	63.0					25000	50000

^a^ Values given as μg/ml, *MIC* ^a^ Minimum Inhibitory Concentration, *MBC* ^a^ Minimal Bactericidal Concentration

**Table 3:** Antimicrobial activity of aqueous extracts from leaf and flower parts of *A. rugosa*

Organisms	Leaf part		Flower part		Penicillin		Gentamycin		Fluconazole	
	MIC^a^	MBC^a^	MIC^a^	MBC^a^	MIC^a^	MBC^a^	MIC^a^	MBC^a^	MIC^a^	MBC^a^
*Staphylococcus aureus*	50	100	100	1000	0.026	0.053				
*Escherichia coli*	50	100	50	100			0.1	0.2		
*Candida albicans*	25	50	100	1000					25	30

^a^ Values given as μg/ml, *MIC* ^a^ Minimum Inhibitory Concentration, *MBC* ^a^ Minimal Bactericidal Concentration

**Table 4:** Antimicrobial activity of alcohol extracts from leaf and flower parts of *A. rugosa*

Organisms	Leaf part		Flower part		Penicillin		Gentamycin		Fluconazole	
	MIC^a^	MBC^a^	MIC^a^	MBC^a^	MIC^a^	MBC^a^	MIC^a^	MBC^a^	MIC^a^	MBC^a^
*Staphylococcus aureus*	25	50	50	100	0.026	0.053				
*Escherichia coli*	50	100	25	50			0.1	0.2		
*Candida albicans*	25	50	50	100					25	30

^a^ Values given as mg/ml, *MIC* ^a^ Minimum Inhibitory Concentration, *MBC* ^a^ Minimal Bactericidal Concentration

Table 2 showed EOF is strong inhibition against *S.aureus* and *E. coli*, strong inhibition against *E. coli* of EOL. And low activity against *B. albicans* from EOF and EOL. The results of MIC, MBC values indicated that *E. coli* was inhibited at lower concentrations by EOL than *S. aureus,* which the lowest MIC value is 9.4 μg/ml. The EOL was sensitive than EOF in fungicidal activity against *B.albicans.* The antibacterial ability showed by the EOL may be due to the chemical composition.

### Cell viability assay (MTT)

**Fig. 4:**
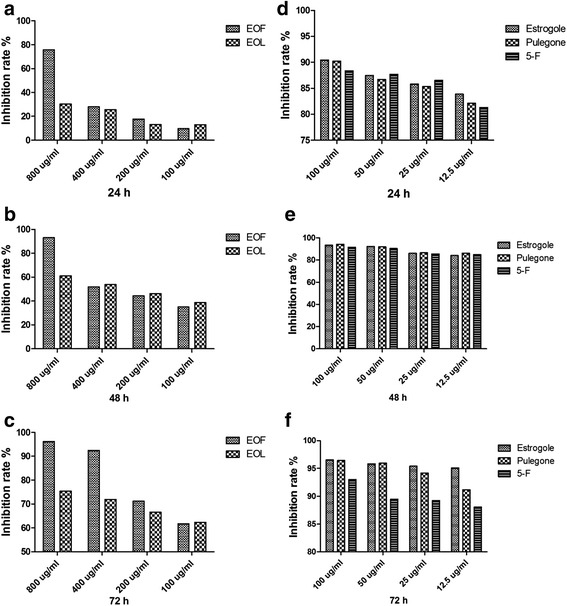
Inhibition rate of each group at different time (24 h, 48 h, 72 h), expressed in percentage

Among the samples for cytotoxicity against SGC-7901 cells, the result of MTT assay showed a dose and time-dependent increase in demage induced by S1-S4 in SGC-7901 cells (Fig. 4). Meanwhile, the EOF showed sensitive (inhibition rate >70%) to SGC-7901 cells ranging from 250 to 1000 ug/ml, from 500 to 1000 ug/ml for the EOL, from 12.5 to 100 ug/ml for estrogole, pulegone and 5-F (inhibition rate >85%). Comparing the inhibition rates, the estrogole and pulegone inhibition effect were better, which were pure and single chemical components. Meanwhile, EOF have better cytotoxicity activity than EOL, which inhibition rate reach to 96.24% at 800 ug/ml (72 h), extracted from cultivated plant of *A. rugosa*.

There were significantly differences in inter-group and intra-group of EOF, according to analysis of variance (*P* < 0.05).

Compared EOF and EOL groups, there was significantly differences in inhibition rates under 800 ug/ml (24, 48, 72 h), and 400 ug/ml (72 h). However, there is no significantly differences in estrogole, pulegone and 5-F groups (*P* > 0.05).

We acquired that EOF and EOL have a certain cytotoxicity activity, especially EOF. Considering plant application value, explore the single and pure chemical components from EOF and EOL, it was found estrogole and pulegone have well inhibition effect. This was potential anti-tumor plant or components, which need to further research and may obtain worthwhile discovery.

## Conclusions

Considering the application value, the characters of microscopic identification of *A. rugosa* should be taken into account as quality control parameters for its pharmacognostic study. With the development of industry and population growth, environmental pollution is also more serious. The forest vegetation is broken seriously, and medicinal herb is severe decreased. So we should rational utilization of medicinal herbs.

Many of the components identified in essential oils have previously been identified from other Lamiaceae species, previously studied by us [12, 13]. In the EOF, 21 components were identified which the major compounds were oxygenated terpenes (35.4%), including pulegone (34.1%), estragole (29.5%), and p-Menthan-3-one (19.2%). Meanwhile, 26 components were identified in EOL, and p-Menthan-3-one (48.8%) and estragole (20.8%) as the main components. The oils have high contents of p-Menthan-3-one (Fig. 2c, Chemical formula, CAS: 1196-31-2).

The experimental plant was cultivated in Xinjiang by our research group, which were collected among 5-7 month for *A. rugosa.* We initiated this study to investigate the antimicrobial activity and cytotoxicity of essential oils of EOL and EOF from *A. rugosa*. We isolated different parts of *A. rugosa* offer choice for clinical treatments and application. Our results thus offer reliable base for resource optimization, clinical choice and standard of quality control.

## Abbreviations

5-F: 5-fluorouracil
*A. rugosa*: Agastache rugosa
ATCC: American Type Culture Collection
*B. albicans*: *Blastomyces albicans*
*C. albicans*: *Candida albicans*
DMSO: Dimethyl sulfoxide
*E. coli*: *Escherichia coli*
EOF: Essential oil of flower
EOL: Essential oil of leaves
FAA: Formalin fixed-acetic acid-alcohol liquid
GC: Gas Chromatography
GC-MS: Gas Chromatography coupled to a Mass Spectrometry
MBC: Minimal bactericidal concentration
MIC: Minimal inhibitory concentration
MTT: 3 -(4,5-dimethyl-2-thiazolyl)-2,5-diphenyl-2-H-tetrazolium bromide
NCCLS: National Committee for Clinical Laboratory Standards
*S. aureus*: *Staphylococcus aureus*
